# Extracellular matrix rigidity modulates neuroblastoma cell differentiation and N-myc expression

**DOI:** 10.1186/1476-4598-9-35

**Published:** 2010-02-10

**Authors:** Wilbur A Lam, Lizhi Cao, Vaibhavi Umesh, Albert J Keung, Shamik Sen, Sanjay Kumar

**Affiliations:** 1 Department of Bioengineering, University of California, Berkeley, USA; 2 Department of Pediatrics, Division of Pediatric Hematology/Oncology, University of California, San Francisco, USA; 3 Department of Chemical Engineering, University of California, Berkeley, USA

## Abstract

Neuroblastoma is a pediatric malignancy characterized by tremendous clinical heterogeneity, in which some tumors are extremely aggressive while others spontaneously differentiate into benign forms. Because the degree of differentiation correlates with prognosis, and because differentiating agents such as retinoic acid (RA) have proven to decrease mortality, much effort has been devoted to identifying critical regulators of neuroblastoma differentiation in the cellular microenvironment, including cues encoded in the extracellular matrix (ECM). While signaling between tumor cells and the ECM is classically regarded to be based purely on biochemical recognition of ECM ligands by specific cellular receptors, a number of recent studies have made it increasingly clear that the biophysical properties of the ECM may also play an important role in this cross-talk. Given that RA-mediated neuroblastoma differentiation is accompanied by profound changes in cell morphology and neurite extension, both of which presumably rely upon mechanotransductive signaling systems, it occurred to us that mechanical cues from the ECM might also influence RA-mediated differentiation, which in turn might regulate clinically-relevant aspects of neuroblastoma biology. In this study, we tested this hypothesis by subjecting a series of neuroblastoma culture models to ECM microenvironments of varying mechanical stiffness and examined the regulatory role of ECM stiffness in proliferation, differentiation, and expression of tumor markers. We find that increasing ECM stiffness enhances neuritogenesis and suppresses cell proliferation. Remarkably, increasing ECM stiffness also reduces expression of N-Myc, a transcription factor involved in multiple aspects of oncogenic proliferation that is used for evaluating prognosis and clinical grading of neuroblastoma. Furthermore, the addition of RA enhances all of these effects for all ECM stiffnesses tested. Together, our data strongly support the notion that the mechanical signals from the cellular microenvironment influence neuroblastoma differentiation and do so synergistically with RA. These observations support further investigation of the role of microenvironmental mechanical signals in neuroblastoma proliferation and differentiation and suggest that pharmacological agents that modulate the underlying mechanotransductive signaling pathways may have a role in neuroblastoma therapy.

## Findings

Inducing tumor cells to differentiate is an important therapeutic goal in cancer, especially in neuroblastoma, one of the few malignancies that demonstrates spontaneous differentiation and regression to a benign state [1]. Previous research has shown that cross-talk between neuroblastoma cells and the extracellular matrix (ECM) influences differentiation [2], and recent studies have identified key genes that regulate those interactions [3,4]. Neuroblastoma differentiation can also be induced pharmacologically with retinoic acid (RA) [5]; indeed, RA has been demonstrated to have significant clinical benefit in neuroblastoma, which has resulted in inclusion of RA in treatment regimens for high risk disease [6-8]. RA-induced signaling in neuroblastoma affects expression of N-Myc and other proto-oncogenes but also has been shown to alter the expression and activity of integrins, Rho GTPases, and the actin cytoskeleton. These molecular components act in concert to sense and process signals from the ECM [9-12] and to mediate cell shape and cytoarchitecture. In addition, altering the biochemical composition of the ECM itself induces morphological differentiation of neuroblastoma cells [13-15]. Together, these data strongly hint that signals encoded in the ECM may play a significant role in guiding NB differentiation.

While the ECM is classically regarded to instruct cell behavior primarily through biochemical recognition by cell adhesion receptors, it has become increasingly clear that another component of ECM-based signaling is fundamentally biophysical in nature. In particular, the mechanical rigidity (stiffness) of the ECM can profoundly alter cellular behavior, including morphology, motility, and proliferation [16-18]. While the mechanisms of this "rigidity sensing" remain incompletely understood, ECM rigidity appears to dictate the amount of stress or strain the cellular cytoskeleton can impose upon adhesion plaques, which is then sensed by specific mechanosensory molecules and regulates both adhesion plaque maturation and adhesion-dependent signaling [19-21]. In fact, many processes relevant to neuroblastoma, such as stem cell differentiation, neuronal maturation, neurite extension, and the malignant potential and phenotype of tumor cells have all been shown to be influenced by ECM stiffness and other biophysical properties [19,22-30]. Interestingly, signaling pathways that underlie this biophysical cross-talk and those implicated in RA-mediated neuritogenesis partially coincide [16,31-34], suggesting that mechanotransductive signals from the ECM may affect neuroblastoma differentiation.

In this study, we tested the hypothesis that mechanical cues from the ECM might influence spontaneous and RA-induced neuroblastoma differentiation and the expression of clinically-relevant markers of cell proliferation and differentiation. We cultured neuroblastoma cells on ECM substrates of various stiffnesses, exposed the cells to defined doses of RA, and measured neurite extension and Ki67 and N-Myc expression. To control ECM stiffness, we fabricated polyacylamide (PA) hydrogels whose stiffness could be controlled by setting the monomer (acrylamide): crosslinker (bis-acrylamide) ratio and which could be rendered suitable for cell adhesion by covalently conjugating collagen I. We and others have previously validated this platform [35,36] and demonstrated that ECM rigidity can be varied independently of biochemical composition and ligand density [29,37,38].

**Figure 1:**
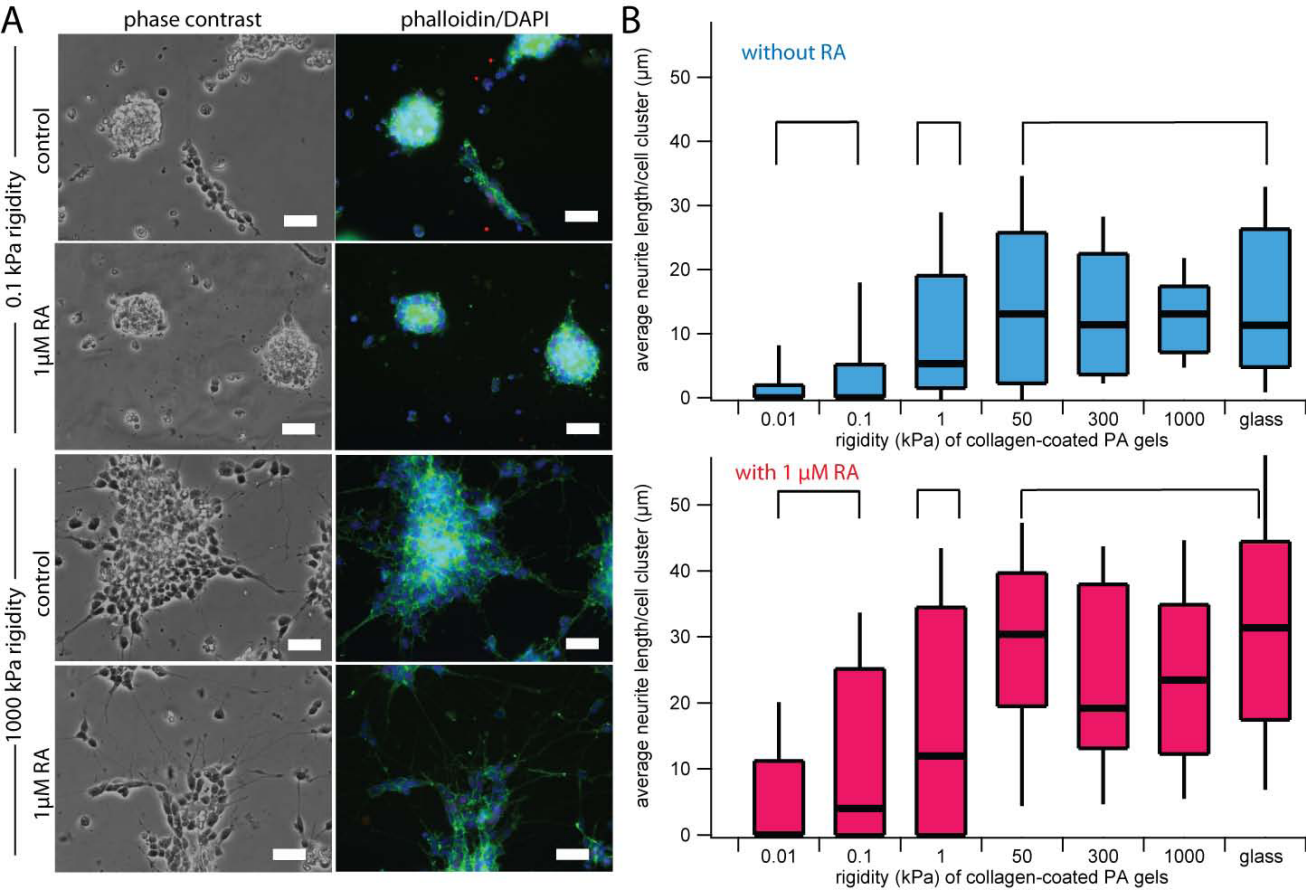
**Extracellular matrix rigidity influences morphological differentiation of neuroblastoma cells**. (A) Phase contrast and fluorescence images of cell nuclei (DAPI) and actin (phalloidin) of SK-N-DZ neuroblastoma cells cultured on 0.1 kPa versus 1000 kPa collagen-coated polyacrylamide (PA) gels with or without exposure to 1 μM 13-cis RA for three days. Scalebar = 50 μm. (B) Average neurite length of SK-N-DZ neuroblastoma cell clusters cultured on collagen-coated PA gels of varying rigidities with and without exposure to 1 μM 13-cis RA. Populations within brackets are statistically indistinguishable from each other but statistically distinguishable from populations in the other brackets (*p *< 0.01 for all significant comparisons). Each population represents >100 cell clusters. 90th percentile, 75th percentile, median, 25th percentile, and 10th percentile values are represented by the top whisker, top line, middle line, bottom line, bottom whisker, respectively, of each bar.

To investigate how ECM rigidity affects morphological differentiation, we cultured SK-N-DZ neuroblastoma cells on collagen I-laminated PA gels of varying rigidities. The stiffnesses of our ECMs overlapped the range of bulk rigidities of *in vivo *tissues, including tissues of origin and metastasis for neuroblastoma (0.01 kPa to 10000 kPa) [39]. Cells exhibited dramatic stiffness-dependent phenotypic differences, with cells cultured on stiffer ECMs coalescing into a few large clusters with extensive actin-rich neurites, which are hallmarks of neuroblastoma differentiation [40]. In contrast, cells cultured on softer ECMs were more homogenously dispersed and exhibited less clustering and neuritogenesis. The addition of 13-cis-RA increased neuritogenesis under all conditions (Figure 1A). Neurite length is used as a morphological index of the extent of neuroblastoma differentiation [9,12,41], and indeed, the average neurite length per cell cluster increased with increasing ECM rigidity (Figure 1B). However, this relationship was not linear across the range of ECM rigidities, as neurite lengths of cells cultured on ECMs with rigidities of 0.01 and 0.1 kPa were statistically indistinguishable from each other but statistically different compared to other groups (*p *< 0.01). Similarly, neurite lengths of cells on ECMs with rigidities from 50 to 10,000 kPa were not statistically different from each other but were statistically different compared to the other groups (*p *< 0.01). Within the range of ECM rigidities we tested, we observed three regimes of neurite lengths at 0.01-0.1 kPa, 1 kPa, and 50-10000 kPa that were statistically distinct from one another. Furthermore, we found that the addition of RA increased neurite length for all rigidities tested and that this increase was independent of substrate stiffness. We observed similar trends with SH-SY5Y and SK-N-SH cell lines, suggesting that this effect is generalizable across multiple neuroblastoma models (Additional file 1).

**Figure 2:**
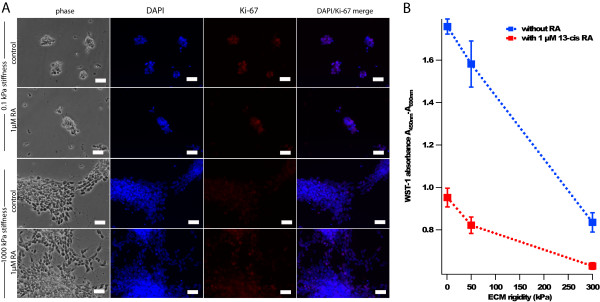
**Extracellular matrix rigidity modulates neuroblastoma cell proliferation**. (A) Phase contrast and fluorescence images of cell nuclei (DAPI) and Ki67 antibody staining (red), a marker of cell proliferation, of SK-N-DZ neuroblastoma cells on 0.1 kPa and 1000 kPa collagen-coated PA gels in the presence or absence of 1 μM 13-cis RA for three days. Scalebar = 50 μm. (B) WST-1 cell proliferation assay of SK-N-DZ neuroblastoma cells cultured on 1, 50, and 300 kPa collagen-coated PA gels with and without exposure to 1 μM 13-cis RA for three days. Error bars represent standard deviations of 5 technical replicates.

To rule out the possibility that ECM stiffness-dependent differences in neurite lengths were solely due to biomechanical facilitation of neurite extension rather than alterations in proliferation and differentiation *per se*, we directly investigated the effect of ECM stiffness on neuroblastoma cell proliferation. When we immunostained SK-N-DZ neuroblastoma cells for the proliferative marker Ki67, we found that cells cultured on softer ECMs showed increased Ki67 staining than cells cultured on stiffer ECMs (Figure 2A). To quantify cell number more rigorously, we measured metabolic activity with the colorimetric mitochondrial reductase substrate WST-1, which revealed an inverse correlation between ECM stiffness and cell proliferation either with or without exposure to RA (r = 0.99 and 0.97, respectively; Figure 2B). Exposure to RA further reduced cell proliferation on both high and low ECM rigidities, consistent with our measurements of neurite length.

**Figure 3:**
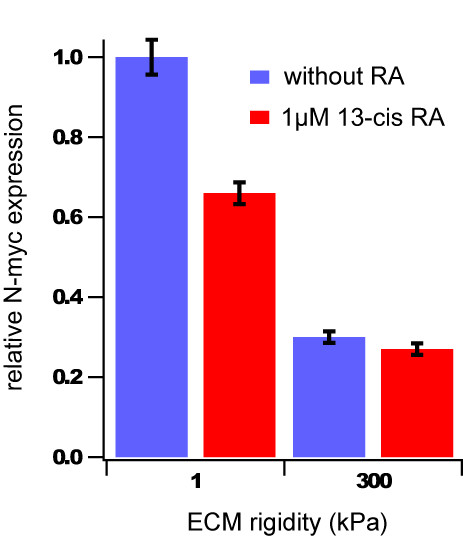
**ECM rigidity modulates N-myc expression in neuroblastoma cells and does so synergistically with retinoic acid**. Quantitative-RT-PCR analysis of relative N-Myc expression in SK-N-DZ neuroblastoma cells cultured on 1.0 kPa versus 300 kPa collagen-coated PA gels in the presence or absence of 1 μM 13-cis RA for three days. Error bars represent standard deviations of 3 technical replicates.

To explore whether ECM rigidity might also influence the expression of markers with clinical prognostic value, we measured the effects of ECM stiffness on the expression of N-Myc, a key transcription factor involved in many aspects of cell proliferation. N-Myc gene amplification is an important clinical prognostic marker in neuroblastoma, and reducing N-Myc expression can induce neuroblastoma differentiation *in vitro *and may improve the prognosis of patients with N-Myc-amplified tumors [1,42-47]. Quantitative RT-PCR analyses using the N-Myc-amplified SK-N-DZ cell line revealed that N-Myc expression decreases with increasing ECM rigidity and that ECM rigidity appears to have a synergistic effect with RA in reducing N-Myc expression (Figure 3).

Signals from the ECM canonically direct cell behavior through biochemical recognition by receptors at the cell surface, which in turn triggers specific signaling networks that alter cell behavior. In this study, we show that *mechanical *signals encoded in the ECM (i.e., ECM rigidity) can modulate neuroblastoma cell behaviors relevant to tumorigenesis. Specifically, we have shown ECM rigidity potentiates neuroblastoma cell differentiation and decreases cell proliferation and N-Myc expression. Furthermore, ECM rigidity appears to act synergistically with RA-induced cell differentiation as evidenced by morphometric analysis and measurements of cell proliferation and N-Myc expression. Further studies are required to elucidate the underlying mechanisms of this phenomenon, but the decreased cell proliferation observed with increasing ECM rigidity may be due in part to the attenuation of N-Myc expression. It is also highly likely that components of the cellular mechanotransductive machinery, such as integrins, adhesion proteins, Rho family GTPases, and cytoskeletal proteins are key players, and further study will be needed to narrow this list. In addition, while our results show that mechanical inputs can control N-Myc expression, it remains to be seen whether this relationship is bidirectional; i.e., whether manipulation of N-Myc expression can control mechanosensitivity. In addition, future experiments involving primary human neuroblastoma cells should help determine to what extent our results with culture models are directly translatable to the clinical disease. While many biochemical and cellular factors have been previously shown to influence neuroblastoma cell differentiation and sensitivity to chemotherapeutic agents, to our knowledge this is the first evidence that a purely biophysical parameter can directly influence biological indicators of neuroblastoma differentiation that correlate with prognosis and sensitivity to a pharmacologic agent.

This study raises novel questions about both neuroblastoma biology and therapy that warrant further investigation. For example, RA is insufficient as a single agent to achieve sustained clinical remission, and our results raise the intriguing prospect that this may be due in part to the absence of additional microenvironmental cues needed to achieve optimal differentiation. In particular, neuroblastoma metastasizes to a wide variety of tissues and it is possible that the diversity of mechanical microenvironments encountered in these tissues might modulate either malignant phenotype or RA chemosensitivity. Interestingly, additional studies revealed that ECM rigidity did not affect neuroblastoma chemosensitivity to doxorubicin (Additional File 2), suggesting that the observed synergy between ECM rigidity and pharmacological agents may not occur with standard chemotherapy but only with differentiation agents such as RA. In addition, our results beg the question of whether pharmacologic modulation of mechanotrandsductive signaling pathways may have a role in neuroblastoma therapy. Finally, the observation that a biophysical signal from the ECM can modulate tumor cell differentiation has implications beyond neuroblastoma, as N-Myc overexpression can contribute to the pathophysiology of other cancers, such as retinoblastoma, small cell lung carcinoma, and glioblastoma [48].

## Materials and methods

### Polyacrylamide gels

Polyacrylamide gels of defined rigidity were synthesized as previously described [29,36].

### Cell lines, stains, pharmacological agents

SK-N-DZ, SH-SY5Y, and SK-N-SH cell lines (ATCC) were cultured on polyacrylamide gels of varying rigidities at 5% CO_2_, 37°C in DMEM/10% FBS with or without 13-cis-RA (Sigma). Neurite length measurements were made via direct visualization with phase-contrast microscopy. Ki67, DAPI, and phalloidin (Invitrogen) fluorescence staining were performed on cells cultured on the gels after 2% paraformaldehyde fixation and 0.1% Triton X (Sigma) permeabilization. For experiments involving Ki67 staining, microscopy settings (exposure time, gain, etc.) remained constant to allow for qualitative comparisons in fluorescence signal across different conditions.

### Statistical analyses

Analyses of variances were used to compare measurements of average neurite lengths per cell cluster on substrates of different rigidities (SPSS). Bonferroni post-hoc analyses were performed to compare each pair of populations.

### RNA extraction and RT-PCR

RNA was extracted from the cells on gels by and converted to DNA using standard Trizol (Invitrogen) and reverse transcriptase (Applied Biosystems) protocols. Gene expression via RT-PCR using the ΔΔCt method was performed using primers for N-myc (Hs01014940_m1 from Applied Biosystems) and GAPDH as a control.

### WST-1 cell proliferation assay

Cells were seeded at the same cell density for all experiments and cultured on ECMs of varying rigidities. After three days, WST-1 reagent (Roche) was added to the medium at 10% final volume and incubated for 2.5 hr. Supernatant was then analyzed per manufacturer's specifications.

## Competing interests

The authors declare that they have no competing interests.

## Authors' contributions

WAL, LC, and SK conceived of and designed the study. WAL, LC, and VU fabricated the gels, conducted cell culture experiments and analysis, and carried out cell proliferation experiments. VU and AJK carried out the qRT-PCR experiments and interpretation. SS carried out the chemosensitivity experiments. WAL, LC, and SK performed the statistical analysis and drafted the manuscript. All authors read and approved the final manuscript.

## Supplementary Material

Additional file 1 **Extracellular matrix rigidity influences morphological differentiation of SH-SY5Y and SK-N-SH neuroblastoma cells**. (A) Average neurite length of SH-SY5Y neuroblastoma cell clusters cultured on collagen-coated PA gels of varying rigidities with and without exposure to 1 μM 13-cis RA. (B) Average neurite length of SK-N-SH neuroblastoma cell clusters cultured on collagen-coated PA gels of varying rigidities with and without exposure to 1 μM 13-cis RA. Populations within brackets are statistically indistinguishable from each other but statistically distinguishable from populations outside those brackets (*p *< 0.05 for all significant comparisons). Each population represents >100 cell clusters. 90th percentile, 75th percentile, median, 25th percentile, and 10th percentile values are represented by the top whisker, top line, middle line, bottom line, bottom whisker, respectively, of each bar. Click here for file

Additional file 2 **ECM rigidity does not affect neuroblastoma chemosensitivity to doxorubicin**. SK-N-DZ neuroblastoma cells were cultured on several collagen-coated PA gels with rigidities of 1 and 300 kPa and then exposed to 1 μM doxorubicin. At 24 hr of exposure to doxorubicin, cells were also exposed to 25 μg/mL of propidium iodide (PI), a fluorescent marker for cell death, and then immediately detached from the PA gels and separated using Accutase and Accumax, respectively (Innovative Cell Technologies). Using flow cytometry, the proportion of live (PI negative) versus dead cells (PI positive) of each cell suspension was then determined. The protocol was repeated at 72 hr of doxorubicin exposure on separate cells cultured on collagen-coated PA gels with the same rigidities. At both 24 hr (A) and 72 hr (B), there was no appreciable difference in the proportion of live versus dead cells between SK-N-DZ neuroblastoma cells cultured on 1 kPa gels versus 300 kPa gels. Click here for file
