# Supplementary material for: Extracellular matrix rigidity modulates neuroblastoma cell differentiation and N-myc expression
Source: Mol Cancer. 2010 Feb 10;9:35. doi: 10.1186/1476-4598-9-35 (PMC2831820; doi:10.1186/1476-4598-9-35)
Supplement: Additional file 1 — Extracellular matrix rigidity influences morphological differentiation of SH-SY5Y and SK-N-SH neuroblastoma cells. (A) Average neurite length of SH-SY5Y neuroblastoma cell clusters cultured on collagen-coated PA gels of varying rigidities with and without exposure to 1 μM 13-cis RA. (B) Average neurite length of SK-N-SH neuroblastoma cell clusters cultured on collagen-coated PA gels of varying rigidities with and without exposure to 1 μM 13-cis RA. Populations within brackets are statistically indistinguishable from each other but statistically distinguishable from populations outside those brackets (p < 0.05 for all significant comparisons). Each population represents >100 cell clusters. 90th percentile, 75th percentile, median, 25th percentile, and 10th percentile values are represented by the top whisker, top line, middle line, bottom line, bottom whisker, respectively, of each bar. [file 1476-4598-9-35-S1.PDF]

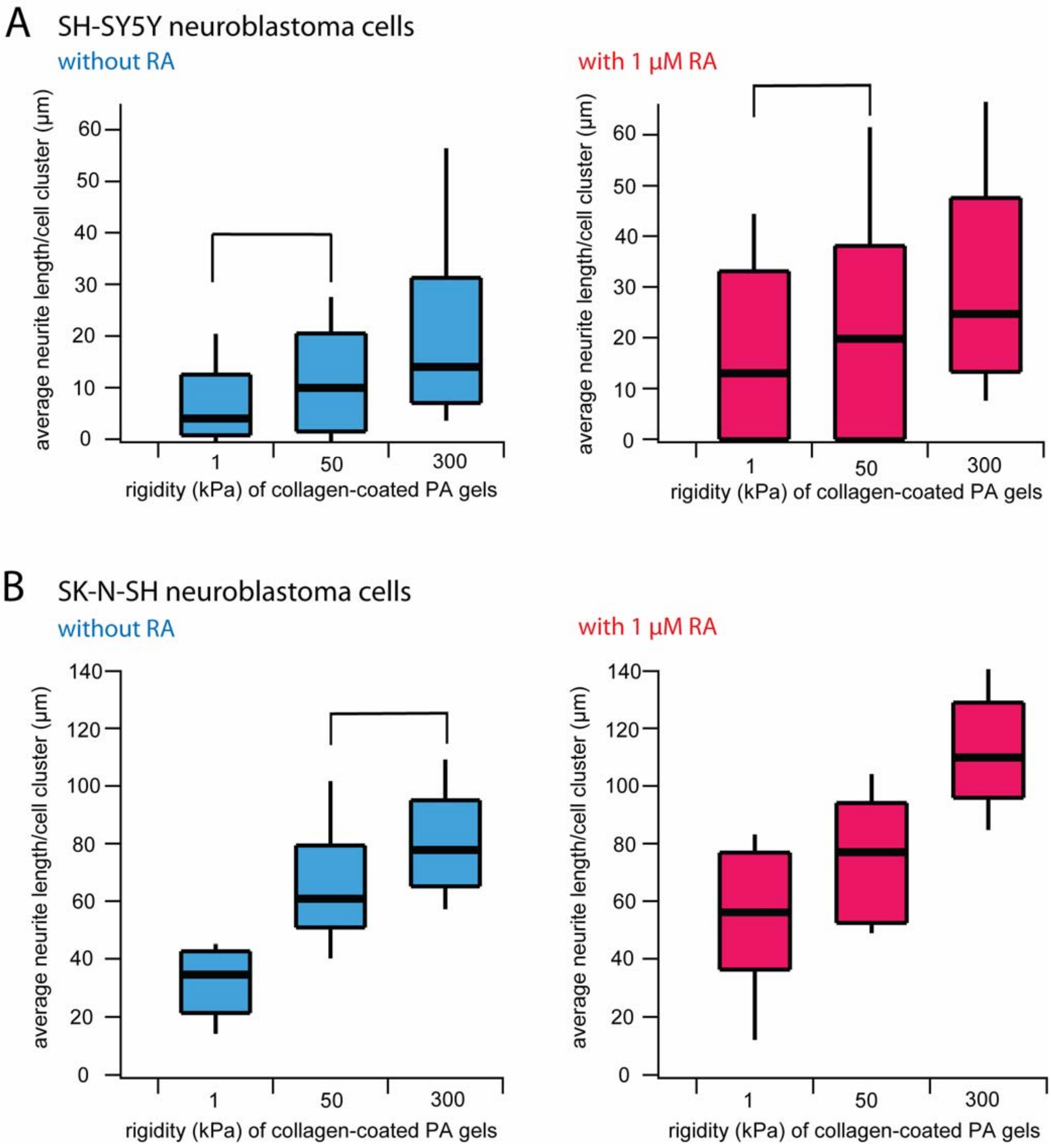

Supplemental Figure 1 – Extracellular matrix rigidity influences morphological differentiation of SH-SY5Y and SK-N-SH neuroblastoma cells. (A) Average neurite length of SH-SY5Y neuroblastoma cell clusters cultured on collagen-coated PA gels of varying rigidities with and without exposure to 1 µM 13-cis RA. (B) Average neurite length of SK-N-SH neuroblastoma cell clusters cultured on collagen-coated PA gels of varying rigidities with and without exposure to 1 µM 13-cis RA. Populations within brackets are statistically indistinguishable from

each other but statistically distinguishable from populations outside those brackets ($p<0.05$ for all significant comparisons). Each population represents >100 cell clusters. 90th percentile, 75th percentile, median, 25th percentile, and 10th percentile values are represented by the top whisker, top line, middle line, bottom line, bottom whisker, respectively, of each bar.
