# Supplementary material for: Extracellular matrix rigidity modulates neuroblastoma cell differentiation and N-myc expression
Source: Mol Cancer. 2010 Feb 10;9:35. doi: 10.1186/1476-4598-9-35 (PMC2831820; doi:10.1186/1476-4598-9-35)
Supplement: Additional file 2 — ECM rigidity does not affect neuroblastoma chemosensitivity to doxorubicin. SK-N-DZ neuroblastoma cells were cultured on several collagen-coated PA gels with rigidities of 1 and 300 kPa and then exposed to 1 μM doxorubicin. At 24 hr of exposure to doxorubicin, cells were also exposed to 25 μg/mL of propidium iodide (PI), a fluorescent marker for cell death, and then immediately detached from the PA gels and separated using Accutase and Accumax, respectively (Innovative Cell Technologies). Using flow cytometry, the proportion of live (PI negative) versus dead cells (PI positive) of each cell suspension was then determined. The protocol was repeated at 72 hr of doxorubicin exposure on separate cells cultured on collagen-coated PA gels with the same rigidities. At both 24 hr (A) and 72 hr (B), there was no appreciable difference in the proportion of live versus dead cells between SK-N-DZ neuroblastoma cells cultured on 1 kPa gels versus 300 kPa gels. [file 1476-4598-9-35-S2.PDF]

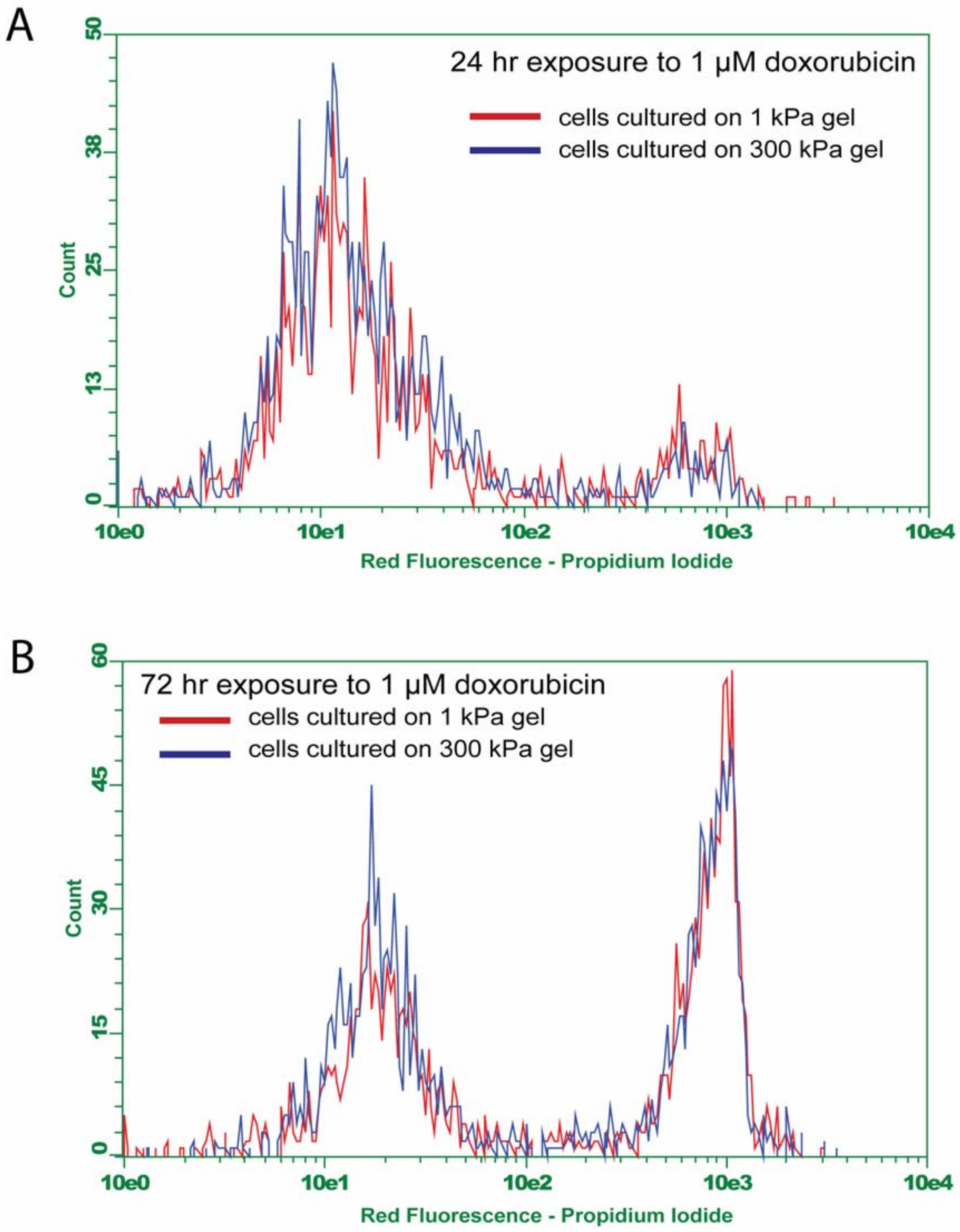

Supplemental Figure 2 - ECM rigidity does not affect neuroblastoma chemosensitivity to doxorubicin. SK-N-DZ neuroblastoma cells were cultured on several collagen-coated PA gels with rigidities of 1 and 300 kPa and then exposed to 1 µM doxorubicin. At 24 hr of exposure to doxorubicin, cells were also exposed to 25 µg/mL of propidium iodide (PI), a fluorescent marker for cell death, and then immediately detached from the PA gels and separated using Accutase and Accumax, respectively (Innovative Cell Technologies). Using flow cytometry, the proportion of live (PI negative) versus dead cells (PI positive) of each cell suspension was then determined. The protocol was repeated at 72 hr of doxorubicin exposure on separate cells cultured on collagen-coated PA gels with

the same rigidities. At both 24 hr (A) and 72 hr (B), there was no appreciable difference in the proportion of live versus dead cells between SK-N-DZ neuroblastoma cells cultured on 1 kPa gels versus 300 kPa gels.
